# Dosimetric Feasibility Study of Dose Escalated Stereotactic Body Radiation Therapy (SBRT) in Locally Advanced Pancreatic Cancer (LAPC) Patients: It Is Time to Raise the Bar

**DOI:** 10.3389/fonc.2020.600940

**Published:** 2020-12-17

**Authors:** Renzo Mazzarotto, Nicola Simoni, Stefania Guariglia, Gabriella Rossi, Renato Micera, Riccardo De Robertis, Alessio Pierelli, Emanuele Zivelonghi, Giuseppe Malleo, Salvatore Paiella, Roberto Salvia, Carlo Cavedon, Michele Milella, Claudio Bassi

**Affiliations:** ^1^Department of Radiation Oncology, University of Verona Hospital Trust, Verona, Italy; ^2^Department of Medical Physics, University of Verona Hospital Trust, Verona, Italy; ^3^Department of Radiology, University of Verona Hospital Trust, Verona, Italy; ^4^Department of General and Pancreatic Surgery, University of Verona Hospital Trust, Verona, Italy; ^5^Department of Oncology, University of Verona Hospital Trust, Verona, Italy

**Keywords:** stereotactic body radiotherapy, pancreatic cancer, locally advanced, dose escalation, ablative dose

## Abstract

**Background and Objective:**

To assess the dosimetric feasibility of a stereotactic body radiotherapy (SBRT) dose escalated protocol, with a simultaneous integrated boost (SIB) and a simultaneous integrated protection (SIP) approach, in patients with locally advanced pancreatic cancer (LAPC).

**Material and Methods:**

Twenty LAPC lesions, previously treated with SBRT at our Institution, were re-planned. The original prescribed and administered dose was 50/30/25 Gy in five fractions to PTV_sib_ (tumor-vessel interface [TVI])/PTV_t_ (tumor volume)/PTV_sip_ (overlap area between PTV_t_ and planning organs at risk volume [PRV_oars_]), respectively. At re-planning, the prescribed dose was escalated up to 60/40/33 Gy in five fractions to PTV_sib_/PTV_t_/PTV_sip_, respectively. All plans were performed using an inspiration breath hold (IBH) technique and generated with volumetric modulated arc therapy (VMAT). Well-established and accepted OAR dose constraints were used (D_0.5cc_ < 33 Gy for luminal OARs and D_0.5cc_ < 38 Gy for corresponding PRV_oars_). The primary end-point was to achieve a median dose equal to the prescription dose for the PTV_sib_ with D_98_≥ 95% (95% of prescription dose is the minimum dose), and a coverage for PTV_t_ and PTV_sip_ of D_95_≥95%, with minor deviations in OAR dose constraints in < 10% of the plans.

**Results:**

PTV_sib_ median (± SD) dose/D_95_/conformity index (CI) were 60.54 (± 0.85) Gy/58.96 (± 0.86) Gy/0.99 (± 0.01), respectively; whilst PTV_t_ median (± SD) dose/D_95_ were 44.51 (± 2.69) Gy/38.44 (± 0.82) Gy, and PTV_sip_ median (± SD) dose/D_95_ were 35.18 (± 1.42) Gy/33.01 (± 0.84) Gy, respectively. With regard to OARs, median (± SD) maximum dose (D_0.5cc_) to duodenum/stomach/bowel was 29.31 (± 5.72) Gy/25.29 (± 6.90) Gy/27.03 (± 5.67) Gy, respectively. A minor acceptable deviation was found for a single plan (bowel and duodenum D_0.5cc_=34.8 Gy). V38 < 0.5 cc was achieved for all PRV luminal OARs.

**Conclusions:**

In LAPC patients SBRT, with a SIB/SIP dose escalation approach up to 60/40/33 Gy in five fractions to PTV_sib_/PTV_t_/PTV_sip_, respectively, is dosimetrically feasible with adequate PTVs coverage and respect for OAR dose constraints.

## Introduction

The results of standard dose radiation therapy (RT) in locally advanced, unresectable pancreatic cancer (LAPC) are unsatisfactory. Conventional RT strategies (conventionally fractionated radiation therapy [CFRT]) have a modest impact on long-term tumor control and survival. Indeed, the randomized LAP-07 Phase III trial failed to demonstrate improvement in overall survival (OS) in LAPC patients by adding CFRT (54 Gy/30 fractions with concurrent capecitabine) after neoadjuvant chemotherapy versus continuation of chemotherapy alone (1). However, RT was associated with a decrease in local progression (32% vs 46%, p = 0.03) without increasing grade ≥ 3 toxicity. Four other randomized trials have compared CFRT, concomitant with chemotherapy, versus chemotherapy alone in LAPC, with interlocutory results: two trials supported a chemo-radiation approach (2, 3), while two did not (4, 5).

Although metastatic disease represents the main cause of morbidity and mortality in LAPC, about one third of patients die from complications related to local tumor progression (6). Moreover, Crane et al. found that local tumor progression was the dominant cause of death in patients alive at more than 15 months (7). Thus, further studies with more effective RT strategies in LAPC are widely expected. In this context, stereotactic body radiation therapy (SBRT) has emerged as an effective component for the multimodal treatment of pancreatic cancer. According to recent studies, SBRT after systemic therapy can increase survival in LAPC compared to either chemotherapy alone or CFRT (8–10). At present, the optimal SBRT schedule has yet to be determined, but the administration of a higher biologically effective dose (BED) is essential to achieve durable tumor control and impact on survival (11). In addition, it can be postulated that SBRT, following induction chemotherapy, may improve the likelihood of resection also for LAPC, in the context of a total neoadjuvant therapy approach (12–14). In particular, the administration of ablative doses to the tumor-vessel interface (TVI), can sterilize the tumor boundaries involving peripancreatic vessels, and together with mass shrinkage, potentially allow surgery.

However, the administration of such high doses is challenging when tumors are close to critical organs at risk (OARs) such as the duodenum, stomach and bowel. A novel approach of SBRT with simultaneous integrated boost (SIB) and simultaneous integrated protection (SIP) has recently been described in an observational study, showing a promising local control (LC) rate of 75% in non-resected patients (versus 82.3% in resected patients; p=0.46) (15). After induction chemotherapy, SBRT was delivered in 5 consecutive daily fractions by administering 30 Gy to the planning target volume tumor (PTV_t_), while simultaneously delivering a 50 Gy SIB to the tumor-vessel interface (PTV_sib_). The SIP volume (PTV_sip_) was created by lowering the dose to 25 Gy on the overlap area between the PTV_t_ and the planning organs at risk volume (PRV_oars_). No acute or late grade ≥ 3 adverse events related to SBRT were observed. Moreover, 34.4% of locally advanced patients received surgical resection. Nonetheless, the performed dosimetric evaluation showed a predominant incidence of in-field failure, with a progression median dose of 40.42 Gy. These data support the need to further investigate the possibility of administering higher doses of RT, using this SBRT approach, in order to improve oncological outcomes in LAPC.

Based on this background, we aimed at performing a dosimetric study to assess the feasibility of SBRT with a SIB/SIP dose escalated protocol in LAPC, to administer higher doses to the tumor, while preserving OAR dose constraints.

## Materials and Methods

### Study Design

Twenty patients with LAPC, treated at our Institution with SBRT in 5 consecutive daily fractions using a SIB/SIP approach (50/30/25 Gy to PTV_sib_/PTV_t_/PTV_sip_, respectively) (15), were re-planned for a dose escalation proposal. Patients were randomly selected from a prospective collected database. The final goal of this dosimetric evaluation study was to escalate the dose up to 60/40/33 Gy in five fractions to PTV_sib_/PTV_t_/PTV_sip_, respectively (**Table 1**). If the planning objectives were not met at this dose level (level IV), the prescription dose would be progressively reduced to the inferior levels (level III, II or I), until the pre-established planning objectives were achieved. The biologically effective dose (BED) was used to compare the different dose levels among each other, and with other recommended fractionations adopted in the clinical practice. The BED was calculated using the linear quadratic formula: BED = *nd* × [1 + *d*/(α/β)], where *n* is the total number of fractions and *d* is the dose per fraction (Gy). Standard α/β ratio for tumors (α/β =10) and normal tissues (α/β =3) was chosen. Dose constraints to organs at risk (OARs) were selected according to recently published guidelines (16). In particular, a D_0.5cc_ < 33 Gy for luminal OARs and a D_0.5cc_ < 38 Gy for corresponding PRVs were adopted.

**Table 1:** SBRT standard dose and levels of SBRT dose escalation proposal*.

	Standard Dose (Gy/fr)	Level I	Level II	Level III	Level IV
**PTV_t_**	30 Gy (6 Gy) BED_10_ 48 Gy	32.5 Gy (6.5 Gy)	35 Gy (7 Gy)	37.5 Gy (7.5 Gy)	**40 Gy (8 Gy)** BED_10_ 72 Gy
**PTV_sib_**	50 Gy (10 Gy) BED_10_ 100 Gy	52.5 Gy (10.5 Gy)	55 Gy (11 Gy)	57.5 Gy (11.5 Gy)	**60 Gy (12 Gy)** BED_10_ 132 Gy
**PTV_sip_**	25 Gy (5 Gy) BED_10_ 37.5 Gy BED_3_ 66.67 Gy	27.5 Gy (5.5 Gy)	30 Gy (6 Gy)	32.5 Gy (6.5 Gy)	***33 Gy (6.6 Gy)***^§^ BED_10_ 54.78 Gy BED_3_ 105.60 Gy

*An increase of 2.5 Gy for the 3 PTVs was planned for each dose level.

^§^The prescribed dose is not increased further in order not to conflict with OARs dose constraints.

### SBRT Protocol and Planning

**Figure 1:**
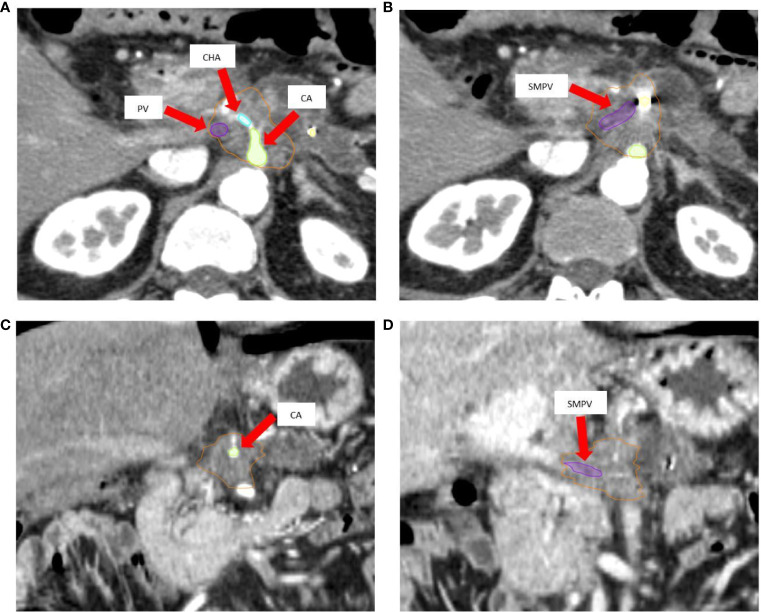
**(A, B)** Axial pancreatic CT-simulation phase images show hypovascular body mass of pancreas, delineated as gross tumor volume (GTV, *orange*) with encasement of celiac axis (CA, *green*), common hepatic artery (CHA, *cyan*), and superior mesenteric-portal venous confluence (SMPV, *violet*). **(C)** Arterial coronal CT-simulation image shows lesion encasing CA. **(D)** Coronal CT-simulation image highlights the SMPV system occlusion and portal vein infiltration.

Patients were immobilized in a supine position with arms over the head, on a custom-made Vac-Lok™ cushion to optimize set-up reproducibility. Fiducial markers (3–4 gold seeds), using an eco-endoscopic procedure (EUS), were placed prior to simulation computed tomography (CT). To manage breathing-induced tumor motion, an inspiration breath hold (IBH) technique was used. Briefly, patients were trained to maintain a regular respiratory cycle, using the real-time position management^®^ system (RPM) (Varian Medical Systems, Palo Alto, CA) as visual guide. At a comfortable inspiration phase of the respiratory cycle, patients were asked to hold their breath (IBH) to allow CT scan acquisition. After a first unenhanced IBH scan, a multi-phase contrast-enhanced simulation CT was performed (**Figure 1**), including the acquisition of an additional 3 to 4 contrast-enhanced IBH scans.

**Figure 2:**
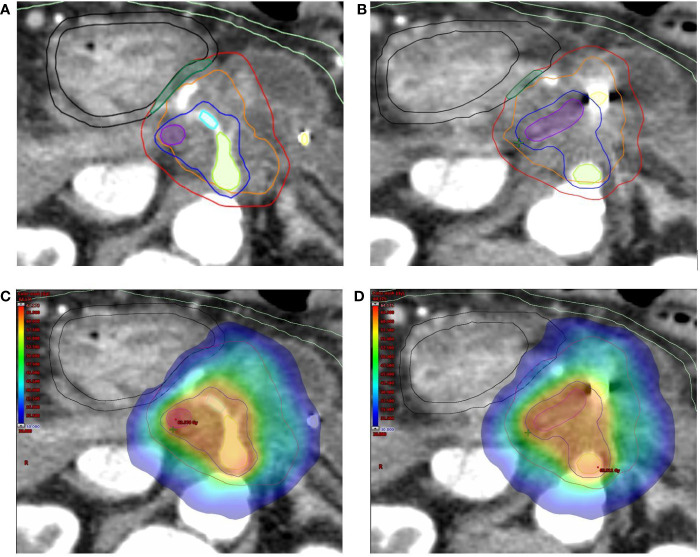
**(A, B)** Target volumes delineation. The high-dose planning target volume (PTV_sib_, *blue*) encompasses the tumor-vessel interface (celiac axis [*green*], common hepatic artery (*cyan*) and superior mesenteric-portal venous confluence [*violet*] + 3 mm expansion) inside the tumor planning target volume (PTV_t_, *red*). Respect to organ at risk constraints is guaranteed by the simultaneous protection volume (PTV_sip_, *dark green*). The following Organ at Risk (OARs) are shown: duodenum (*black*), and bowel (*light green*), as well as the fiducial markers (*yellow*). The Planning at Risk Volume (PRV_oars_) are generated by 3 mm expansion from corresponding OARs (shown with same color on axial images). **(C, D)** Typical dose distribution (color wash) for SBRT plan with Simultaneous Integrated Boost (SIB) and Simultaneous Integrated Protection (SIP). The prescription dose is 60/40/33 Gy in 5 daily fractions to PTV_sib_/PTV_t_/PTV_sip_, respectively. The sample plan demonstrates excellent PTVs coverage with appropriate respect of OARs.

An integrated gross tumor volume (_i_GTV) was defined as the envelope of the GTVs delineated on each CT scan. An _i_GTV-to-PTV margin of 3 mm was applied to generate the PTV tumor (PTV_t_). For critical OARs such as the duodenum, stomach and bowel, a 3 mm expansion PRV_oars_ was defined. The simultaneous protection volume (PTV_sip_) was generated by the intersection of the PTVt and the PRV_oars_. A PTV high dose (PTV_sib_) was generated to encompass the tumor-vessel interface (TVI). Critical vessels (e.g. superior mesenteric artery/vein, portal vein, celiac artery) inside the _i_GTV were contoured for the whole circumference and then expanded by 3 mm to generate the PTV_sib_. If necessary, this PTV_sib_ was contracted to respect a minimal distance of 5 mm from the PTV_sip_ (**Figures 2A, B**).

All SBRT plans were calculated for a TrueBeam^®^ medical linac (Varian Medical System, Palo Alto, CA) equipped with a high definition multileaf collimator (HDMLC-120) and using a Volumetric Modulated Arc Therapy (VMAT) technique (**Figures 2C, D**). A photon energy of 6MV, flattening filter free (FFF) technique, dose rate 1400 MU/min, three arc configuration and anisotropic analytic algorithm (AAA) were used for planning and dose calculation in the Eclipse^®^ treatment planning system (Varian Medical Systems, Palo Alto, CA). All the plans have been calculated using the standard inverse optimization process, based on Dose-Volume Histogram (DVH) parameters.

All the plans were prepared to be managed, during the delivery phase, using an IBH respiratory gating system (RPM^®^ Varian Medical Systems, Palo Alto, CA) with daily IBH cone-beam CT (CBCT) image registration. IBH-CBCTs acquisition allows high quality daily scans with minimized motion artefacts, that, along with the presence of fiducial markers, improves the day-to-day target position verification and reduces inter and intra-fractions errors.

### Study End-Points

The main objective of the study was to ensure adequate coverage of the PTV_sib_, simultaneously respecting dose constraints to OARs. In particular, coverage goals for the targets were:

-median dose equal (± 2%) to the prescription dose for PTV_sib_
-D_98_≥95% (95% of prescription dose is the minimum dose) for PTV_sib_
-maximum point dose of 107% inside PTV_sib_
-D_95_≥95% for PTV_t_ and PTV_sip_

The goal for OARs was a minor deviation in dose constraints in < 10% of the plans.

Dose volume histograms (DVHs) were generated for each plan, and multiple dosimetric parameters for PTVs (PTV_t_, PTV_sib_, and PTV_sip_) and OARs (duodenum, stomach, small and large bowel, spinal cord, liver, kidneys, and PRVs) were evaluated. The conformity index (CI) was defined as the volume encompassed by the 95% isodose divided by the PTV volume. CI was evaluated for PTV_sib_ alone, since this index is formulated based on the paradigm of uniform dose prescription, which is not the case for this SBRT treatment with SIB/SIP approach.

## Results

### Study Population

Baseline characteristics of the 20 patients included in this study are outlined in **Table 2**. All patients had locally advanced pancreatic carcinoma (LAPC), and were considered unresectable due to vascular involvement.

**Table 2:** Baseline characteristics of locally advanced pancreatic cancer patients (n = 20).

**Age, y, median (range)**	65 (39–73)
**Gender, male, n (%)**	13 (65)
**Tumor diameter (mm), (median, min-max)**	31 (20–51)
**Primary Site**	
Head, n (%)	13 (65)
Body, n (%)	7 (35)
**Pre-SBRT chemotherapy**	
Gemcitabine + nab-Paclitaxel, n (%)	11 (55)
FOLFIRINOX, n (%)	9 (45)
**Biliary Stent, yes, n (%)**	7 (35)
**Ca 19-9 values (U/ml)**	
At diagnosis, mean (SD)	601 (± 328)
Pre-SBRT, mean (SD)	54 (± 60)
**Involved vessels**	
CA involvement, n (%)	9 (45)
CHA involvement, n (%)	7 (35)
SMA involvement, n (%)	11 (55)
PV/SMV involvement, n (%)	8 (40)

FOLFIRINOX, fluorouracil, leucovorin, oxaliplatin,

CA, celiac artery; CHA, common hepatic artery; SMA, superior mesenteric artery; PV, portal vein; SMV, superior mesenteric vein.

### SBRT Planning and PTVs Coverage

All the SBRT plans met the predetermined target coverage objectives. **Table 3** describes the results of the treatment plan analysis of the dose escalation proposal (60/40/33 Gy in five fractions to PTV_sib_/PTV_t/_PTV_sip_, respectively). PTV_sib_ median dose, D_95_, and CI were 60.54 Gy (± SD 0.85), 58.96 Gy (± SD 0.85), and 0.99 (± SD 0.01), respectively. The median dose was 44.51 Gy (± SD 2.69) for PTV_t_, and 35.18 Gy (± SD 1.42) for PTV_sip_. For PTV_sib,_ a D_100_≥95% was reached in 18 (90%) plans, while D_98_≥95% was obtained in all cases (100%). A maximum dose of less than 107% for PTV_sib_ was maintained in every plan.

**Table 3:** Treatment plan analysis for PTVs*.

	Dosimetric parameters	Objectives	Results, Mean (± SD)
**PTV_sib_**	Median volume^§^		8.1 cc (5.3–41.2)
	D_median_	60 Gy (± 2%)	60.54 (± 0.85) Gy
	D_max_	<107%	103.7 (± 1.25) %
	D_98_	≥95%	97.5 (± 1.00) %
	D_95_	≥95%	100%
	D_2_	-	61.20 (± 1.25) Gy
	CI	1	0.99 (± 0.01)
**PTV_t_**	Median volume^§^		42.5 cc (21.3–118)
	D_median_	-	44.51 (± 2.69) Gy
	D_95_	≥95%	96 (± 0.80) %
	D_2_	-	59.39 (± 0.93) Gy
**PTV_sip_**	Median volume^§^		5.9 cc (2.2–80.9)
	D_median_	-	35.18 (± 1.42) Gy
	D_95_	≥95%	100%
	D_2_	-	39.81 (± 1.13) Gy

*60/40/33 Gy in five daily fractions to PTV_sib_/PTV_t_/PTV_sip_, respectively.

^§^Median volume (range).

### OAR Constraints

**Figure 3:**
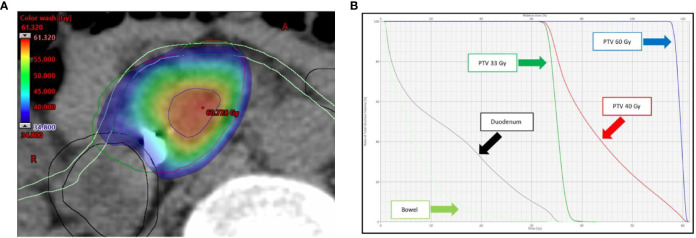
**(A)** Dose distribution (color wash) and **(B)** dose-volume histogram (DVH) for the single SBRT plan showing a minor acceptable deviation for bowel and duodenum (D_0.5cc_ = 34.8 Gy). As shown in panel A, the anatomy for this lesion is rather unfavorable, with the simultaneous protection volume (PTV_sip_, *dark green*), surrounded for almost two thirds of the circumference by the PRVs. The following structures are shown: tumor planning target volume (PTV_t_, *red*), high-dose planning target volume (PTV_sib_, *blue*), duodenum (*black*), and bowel (*light green*). The Planning at Risk Volume (PRV_oars_) are shown with same color on axial image.

With regard to OARs, mean maximum dose (D_0.5cc_) to duodenum/stomach/bowel was 29.31 Gy (± SD 5.72)/25.29 Gy (± SD 6.90)/27.03 Gy (± SD 5.67), respectively. **Table 4** describes treatment plans analysis for OARs. A minor acceptable deviation was observed in a single plan, with bowel and duodenum D_0.5cc_ = 34.8 Gy (**Figure 3**). V38 < 0.5 cc was achieved for all PRV luminal OARs.

**Table 4:** Treatment plans analysis for OARs*.

Organ	Parameter	Constraints	Minor Variation	Major Variation	Results (mean ± SD)
Duodenum	Dmax (0.5 cc) V30_Gy_	<33 Gy <5 cc	≤35 Gy 5–10 cc	>35 Gy >10 cc	29.31 ± 5.72
Stomach	Dmax (0.5 cc) V30_Gy_	<33 Gy <5 cc	≤35 Gy 5–10 cc	>35 Gy > 10 cc	25.29 ± 6.90
Bowel°	Dmax (0.5 cc) V30_Gy_	<33 Gy <5 cc	≤35 Gy 5–10 cc	>35 Gy >10 cc	27.03 ± 5.67
PRV duodenum	Dmax (0.5 cc)	<38 Gy	38–40 Gy	>40 Gy	33.68 ± 6.51
PRV stomach	Dmax (0.5 cc)	<38 Gy	38–40 Gy	>40 Gy	29.51 ± 8.01
PRV bowel	Dmax (0.5 cc)	<38 Gy	38–40 Gy	>40 Gy	32.31 ± 4.37
PRV spinal cord	Dmax	<20 Gy	≤25 Gy	>25 Gy	12.83 ± 2.00
Liver	V12_Gy_	<40%	≤50%	>50%	5.42 ± 5.3
Kidneys (combined)	V12_Gy_	<25%	<25%	>25%	5.34 ± 5.5

SD, standard deviation; cc, cube centimeter; Gy, gray; PRV, planning organ at risk volume.

*60/40/33 Gy in 5 daily fractions to PTV_sib_/PTV_t_/PTV_sip_, respectively.

°Small and large bowel.

## Discussion

Stereotactic body radiation therapy (SBRT) has demonstrated promising results in locally advanced, unresectable pancreatic cancer (LAPC). However, durable local control (LC) remains challenging, and higher biologically effective doses (BED_10_) are suggested to achieve tumor ablation. Advances in radiation delivery techniques, image-guidance (IGRT) and treatment planning, may allow for dose escalation to levels not previously achievable, potentially improving LC and survival. The results of the present study demonstrate that for LAPC, a 5-fraction SBRT with a SIB/SIP dose escalation protocol up to 60/40/33 Gy to PTV_sib_/PTV_t_/PTV_sip_, respectively, is dosimetrically feasible with adequate PTVs coverage and respect for OAR dose constraints.

The clinical rationale for this dosimetric study derives from our current experience with SBRT in pancreatic cancer. In a series of 59 patients treated with SBRT with SIB/SIP at our Institution, we found no G3 toxicities, but a predominant incidence of in-field failures (15). Based on these results, we evaluated the opportunity and feasibility of a dose escalation protocol, with the aim of improving the clinical outcomes of the aforementioned SBRT approach. In this regard, a recent MDACC study provided a remarkable roadmap to achieve a dose escalation up to 60 Gy in SBRT for LAPC (17).

The currently recommended dose in pancreatic SBRT is 33–40 Gy in five fractions (BED_10_ = 54.78–72 Gy) (18), instead BED_10_ of not less than 100 Gy is generally advocated to maximize the RT therapeutic effect and improve oncological outcomes (19). These doses are presumably necessary if the goal of SBRT is to achieve results comparable to surgery. However, when SBRT is applied to pancreatic tumors, the prescription of such high doses is challenging due to the proximity of critical OARs (e.g. duodenum, stomach and bowel), and serious late toxicity, such as perforation, stenosis, and ulcer with bleeding, could be expected (14). In our experience, the use of the SIP in pancreatic SBRT had presumably prevented serious damage to OARs. Considering that less than 10% of patients with LAPC are suitable for surgery after neoadjuvant therapy (20), the administration of such high doses should be as safe as possible. Thus, the use of this 3-dose level SBRT approach can certainly allow to maximize the therapeutic window. Indeed, a clinically acceptable plan was obtained for all patients, with an excellent PTV_sib_ coverage (D_98_≥95% reached in all plans) and adequate respect for OAR dose constraints (a minor acceptable deviation was observed in a single plan for bowel and duodenum D_0.5cc_ = 34.8 Gy), even when the prescribed dose corresponds to 60 Gy and 33 Gy to the TVI and SIP volume, respectively. Noteworthy, the median dose of PTV tumor (PTV_t_) at level IV of the dose escalation proposal was 44.51 (± 2.69) Gy, corresponding to a BED_10_ of 84.13 (± 2.83) Gy. Therefore, as a consequence of the high dose gradient within the tumor target, the PTV_t_ absorbed dose would be consistently higher than the one expected (40 Gy for a BED_10_ of 72 Gy), potentially further increasing the final local effect of the SBRT.

Organ motion control is crucial for a dose escalation proposal. Our standard approach involves the use of the abdominal compressor (15). Indeed, in a study evaluating the effect of abdominal compression in pancreatic cancer, it was observed that with the use of this technique a cranio-caudal (CC) margin of 5 mm was adequate to encompass the tumor target motion in more than 90% of the patients (21). More recently, Campbell et al. compared compression and gating for pancreatic SBRT: the average motion in CC direction was 8.5 mm with abdominal compression, and 5.5 mm with respiratory gating (22). Similarly, the use of a breath hold technique can minimize the required PTV expansion compared with treatment during free breathing or with the use of abdominal compression (23). As a whole, these results suggest that respiratory gating and breath hold may be the best choice for organ motion management in pancreatic SBRT, in particular if a dose escalated approach is planned. Nevertheless, pancreatic region‐dependent variations in respiratory induced organ motion, and their effects on motion control approach, have been described (24). In particular, motion mitigation techniques resulted less effective in the tail region, with no difference between the use of abdominal compression versus respiratory gating, probably due to the larger positional error in the tail region based on the abdominal wall surrogate. Taking this into consideration, in the present study no tail lesions were included for the dose escalated proposal, hence the results are not applicable to the tumors of this pancreatic region. In the near future, the use of Magnetic Resonance-guided Radiation Therapy (MRgRT) will allow a daily online adaptation of the treatment plan, immediately before each fraction delivery, to optimize the dose distribution based on target and OAR anatomy, as well as a real-time management of the organ motion (25).

Another point of discussion is SBRT target delineation. Recent guidelines provide a clear definition for the primary GTV and tumor-vessel interface (TVI), aiming to standardize treatment volumes (16). Moreover, in our and other experiences reported in the literature, a SIB technique was used for clinical dose painting to deliver higher doses to a specific area of the tumor (15, 26–31). **Table 5** summarizes studies describing the use of SBRT with a SIB approach in pancreatic cancer, underlining the variability among authors in the definition of the SIB volume. In the present study, the PTV_sib_ was generated to encompass the TVI, in order to simplify the comparison with the plans evaluated and approved in our clinical practice. Furthermore, the vascular encasement, represented by the TVI is the main obstacle to plan and achieve a curative resection for LAPC. Since the SBRT technique can be easily integrated into a total neoadjuvant therapy (TNT), an ablative boost to the TVI could maximize the possibility of a conversion to surgery. In this regard, to better define TVI, the integration of MRI images with a contrast-enhancement CT-simulation scan, could offer a higher definition of tumor relationship with neighboring vessels and a greater accuracy of target delineation (32).

**Table 5:** Studies using SBRT with Simultaneous Integrated Boost (SIB) for pancreatic cancer.

Reference	Fractions (n)	PTV definition	PTV dose	SIB target	SIB dose
**Chuong et al. (**26**)**	5	PTV = entire tumor + 3–5 mm	25 Gy	TVI (region of vessel abutment/encasement)	35 Gy
**Mellon et al. (**27**)**	5	PTV = GTV (plus motion) + 3–5 mm	30 Gy	TVI (areas of vessel involvement by tumor)	40 Gy
**Shaib et al. (**28**)**	3	PTV = GTV with at-risk area of microscopic spread + 5 mm	36 Gy	PM = volume between the posterior 1 cm of GTV and mesenteric vessel/retroperitoneal soft tissue	45 Gy
**Holyoake et al. (**29**)**	5	PTV = entire tumor + 5 mm	35 Gy	TVI (margin-directed boost)	50 Gy
**Kharofa et al. (**30**)**	5	PTV elective = PTV + customized nodal space and mesenteric vessels	25 Gy	PTV = GTV + TVI	33 Gy
**Koay EJ et al. (**31**)**	5	PTV = (GTV + TVI) + 3 mm	33 Gy	PTV_high_ = PTV with PRV OARs subtracted	50 Gy
**Simoni et al. (**12**)**	5	PTV = entire tumor + 5 mm	30 Gy	TVI (region of vessel abutment/encasement)	50 Gy

n, number; PTV, planning target volume; SIB, simultaneous integrated boost; Gy, gray; TVI, tumor-vessel interface; GTV, gross tumor volume; PM, posterior margin; PRV OARs, planning organs at risk volume.

This study has potential limitations. The sample size is relatively small, and although a reasonable variety of locally advanced diseases was included, not all possible tumor characteristics and anatomical heterogeneity were represented. Therefore, some patients who meet the inclusion criteria for this dose escalated SBRT in the “dosimetric reality”, may not be suitable for the same dose escalation in “real life”. Furthermore, the dose constraints used are based on a commonly accepted consensus for SBRT, however validation in the clinical practice is necessary, thus the inclusion of patients in a clinical trial is strongly recommended. Finally, not all LAPC patients are candidates for SBRT. Exclusion criteria for SBRT are usually as follows: tumor > 6 cm in greatest dimension, nodal spread that cannot be included in the SBRT target volume, and tumors infiltrating the stomach or duodenum. For these patients, an alternative 15-fractions hypofractionated ablative radiation therapy approach may be investigated (**Supplementary Material**).

In conclusion, this study demonstrates that a SBRT dose escalation protocol with a SIB/SIP approach for LAPC up to 60/40/33 Gy in five fractions to PTV_sib_/PTV_t_/PTV_sip_, respectively, is feasible with adequate target coverage and without unacceptable increased OAR exposure. Based on this dosimetric analysis, a Phase II dose escalated trial is ongoing at our Institution.

## Data Availability Statement

The datasets presented in this article are not readily available because the datasets generated for this study are available on request to the corresponding author, subject to approval by the Institutional Review Board. Requests to access the datasets should be directed to nicola.simoni@aovr.veneto.it.

## Ethics Statement

The studies involving human participants were reviewed and approved by Comitato etico per la Sperimentazione Clinica (CESC) delle Province di Verona e Rovigo. Written informed consent for participation was not required for this study in accordance with the national legislation and the institutional requirements.

## Author Contributions

Conceptualization, RMa and NS. Methodology, NS and SG. Validation, GR, RMi, GM, and SP. Plan study and preparation, SG, AP, and EZ. Plan evaluation, NS, GR, and RMi. Writing—original draft preparation, NS and SG. Writing—review and editing, RMi, EZ, and CC. Images curation: NS and RD. Supervision, RMa, RS, MM, and CB. All authors contributed to the article and approved the submitted version.

## Conflict of Interest

The authors declare that the research was conducted in the absence of any commercial or financial relationships that could be construed as a potential conflict of interest.

The handling editor declared a past co-authorship with several of the authors RM, NS.

## References

[1] Hammel P Huguet F van Laethem JL Goldstein D Glimelius B Artru P Effect of Chemoradiotherapy vs chemotherapy on survival in patients with locally advanced pancreatic Cancer controlled after 4 months of gemcitabine with or without Erlotinib: the LAP07 randomized clinical trial. JAMA (2016) 315:1844–53. 10.1001/jama.2016.4324 27139057

[2] Gastrointestinal Tumor Study Group Treatment of locally unresectable carcinoma of the pancreas: comparison of combined-modality therapy (chemotherapy plus radiotherapy) to chemotherapy alone. J Natl Cancer Inst (1988) 80:751–5. 10.1093/jnci/80.10.751 2898536

[3] Loehrer PJ Sr Feng Y Cardenes H Wagner L Brell JM Cella D Gemcitabine alone versus gemcitabine plus radiotherapy in patients with locally advanced pancreatic cancer: an Eastern Cooperative Oncology Group trial. J Clin Oncol (2011) 29:4105–12. 10.1200/JCO.2011.34.8904 PMC3525836 21969502

[4] Klaassen DJ MacIntyre JM Catton GE Engstrom PF Moertel CG Treatment of locally unresectable cancer of the stomach and pancreas: a randomized comparison of 5-fluorouracil alone with radiation plus concurrent and maintenance 5-fluorouracil—an Eastern Cooperative Oncology Group study. J Clin Oncol (1985) 3:373–8. 10.1200/JCO.1985.3.3.373 3973648

[5] Chauffert B Mornex F Bonnetain F Rougier P Mariette C Bouché O Phase III trial comparing intensive induction chemoradiotherapy (60 Gy, infusional 5-FU and intermittent cisplatin) followed by maintenance gemcitabine with gemcitabine alone for locally advanced unresectable pancreatic cancer. Definitive results of the 2000–01 FFCD/SFRO study. Ann Oncol (2008) 19:1592–9. 10.1093/annonc/mdn281 18467316

[6] Iacobuzio-Donahue CA Fu B Yachida S Luo M Abe H Henderson CM DPC4 gene status of the primary carcinoma correlates with patterns of failure in patients with pancreatic cancer. J Clin Oncol (2009) 27:1806–13. 10.1200/JCO.2008.17.7188 PMC2668706 19273710

[7] Crane CH Varadhachary GR Yordy JS Staerkel GA Javle MM Hobbs BD Phase II trial of cetuximab, gemcitabine, and oxaliplatin followed by chemoradiation with cetuximab for locally advanced (T4) pancreatic adenocarcinoma: correlation of Smad4(Dpc4) immunostaining with pattern of disease progression. J Clin Oncol (2011) 29:3037–43. 10.1200/JCO.2010.33.8038 PMC3157965 21709185

[8] de Geus SWL Eskander MF Kasumova GG Ng SC Kent TS Mancias JD Stereotactic body radiotherapy for unresected pancreatic cancer: A nationwide review. Cancer (2017) 123:4158–67. 10.1002/cncr.30856 28708929

[9] Zhong J Patel K Switchenko J Cassidy RJ Hall WA Gillespie T Outcomes for patients with locally advanced pancreatic adenocarcinoma treated with stereotactic body radiation therapy versus conventionally fractionated radiation. Cancer (2017) 123:3486–93. 10.1002/cncr.30706 PMC5589506 28493288

[10] Tchelebi LT Lehrer EJ Trifiletti DM Sharma NK Gusani NJ Crane CH Conventionally fractionated radiation therapy versus stereotactic body radiation therapy for locally advanced pancreatic cancer (CRiSP): An international systematic review and meta-analysis. Cancer (2020) 126:2120–31. 10.1002/cncr.32756 32125712

[11] Arcelli A Guido A Buwenge M Simoni N Mazzarotto R Macchia G Higher Biologically Effective Dose Predicts Survival in SBRT of Pancreatic Cancer: A Multicentric Analysis (PAULA-1). Anticancer Res (2020) 40:465–72. 10.21873/anticanres.13975 31892602

[12] Rajagopalan MS Heron DE Wegner RE Zeh HJ Bahary N Krasinskas AM Pathologic response with neoadjuvant chemotherapy and stereotactic body radiotherapy for borderline resectable and locally-advanced pancreatic cancer. Radiat Oncol (2013) 8:254. 10.1186/1748-717X-8-254 24175982 PMC4228466

[13] Moningi S Dholakia AS Raman SP Blackford A Cameron JL Le DT The Role of Stereotactic Body Radiation Therapy for Pancreatic Cancer: A Single-Institution Experience. Ann Surg Oncol (2015) 22:2352–8. 10.1245/s10434-014-4274-5 PMC4459890 25564157

[14] Suker M Nuyttens JJ Eskens FALM Haberkorn BCM Coene PPLO Van Der Harst E Efficacy and feasibility of stereotactic radiotherapy after folfirinox in patients with locally advanced pancreatic cancer (LAPC-1 trial). EClinicalMedicine (2019) 19(17):100200. 10.1016/j.eclinm.2019.10.013 PMC6933188 31891135

[15] Simoni N Micera R Paiella S Guariglia S Zivelonghi E Malleo G Hypofractionated Stereotactic Body Radiation Therapy With Simultaneous Integrated Boost and Simultaneous Integrated Protection in Pancreatic Ductal Adenocarcinoma. Clin Oncol (R Coll Radiol) (2020) S0936–6555(20)30275-2. 10.1016/j.clon.2020.06.019 32682686

[16] Oar A Lee M Le H Hruby G Dalfsen R Pryor D Australasian Gastrointestinal Trials Group (AGITG) and Trans-Tasman Radiation Oncology Group (TROG) Guidelines for Pancreatic Stereotactic Body Radiation Therapy (SBRT). Pract Radiat Oncol (2020) 10:e136–46. 10.1016/j.prro.2019.07.018 31761541

[17] Colbert LE Rebueno N Monigi S Beddar S Sawakuchi GO Herman JM Dose escalation for locally advanced pancreatic cancer: How high can we go? Adv Radiat Oncol (2018) 3:693–700. 10.1016/j.adro.2018.07.008 30370371 PMC6200902

[18] Palta M Godfrey D Goodman KA Hoffe S Dawson LA Dessert D Radiation Therapy for Pancreatic Cancer: Executive Summary of an ASTRO Clinical Practice Guideline. Pract Radiat Oncol (2019) 9:322–32. 10.1016/j.prro.2019.06.016 31474330

[19] Bernard V Herman JM Pancreas SBRT: Who, What, When, Where, and How…. Pract Radiat Oncol (2020) 10:183–5. 10.1016/j.prro.2019.11.005 31760165

[20] Maggino L Malleo G Marchegiani G Viviani E Nessi C Ciprani D Outcomes of Primary Chemotherapy for Borderline Resectable and Locally Advanced Pancreatic Ductal Adenocarcinoma. JAMA Surg (2019) 154:942. 10.1001/jamasurg.2019.2278 31339530 PMC6659151

[21] Lovelock DM Zatcky J Goodman K Yamada Y Dawson LA Dessert D The effectiveness of a pneumatic compression belt in reducing respiratory motion of abdominal tumors in patients undergoing stereotactic body radiation. Technol Cancer Res Treat (2004) 13:259–67. 10.1016/j.prro.2019.06.016 24206202

[22] Campbell WG Jones BL Schefter T Goodman KA Miften M An evaluation of motion mitigation techniques for pancreatic SBRT. Radiother Oncol (2017) 124:168–73. 10.1016/j.radonc.2017.05.013 PMC5523845 28571887

[23] Forbang Teboh R Srinivasan S Ng SP Aliru ML Herman JM Setup Management for Stereotactic Body Radiation Therapy of Patients With Pancreatic Cancer Treated via the Breath-Hold Technique. Pract Radiat Oncol (2020) 10:e280–9. 10.1016/j.prro.2019.10.012 31669403

[24] Fujimoto K Shiinoki T Yuasa Y Onizuka R Yamane M Evaluation of the effects of motion mitigation strategies on respiration-induced motion in each pancreatic region using cine-magnetic resonance imaging. J Appl Clin Med Phys (2019) 20:42–50. 10.1002/acm2.12693 31385418 PMC6753735

[25] Placidi L Romano A Chiloiro G Cusumano D Boldrini L Cellini F On-line adaptive MR guided radiotherapy for locally advanced pancreatic cancer: Clinical and dosimetric considerations. Tech Innov Patient Support Radiat Oncol (2020) 15:15–21. 10.1016/j.tipsro.2020.06.001 32642565 PMC7334416

[26] Chuong MD Springett GM Freilich JM Park CK Weber JM Mellon EA Stereotactic body radiation therapy for locally advanced and borderline resectable pancreatic cancer is effective and well tolerated. Int J Radiat Oncol Bio Phys (2013) 86:516–22. 10.1016/j.ijrobp.2013.02.022 23562768

[27] Mellon EA Hoffe SE Springett GM Frakes JM Strom TJ Hodul PJ Long-term outcomes of induction chemotherapy and neoadjuvant stereotactic body radiotherapy for borderline resectable and locally advanced pancreatic adenocarcinoma. Acta Oncol (2015) 54:979–85. 10.3109/0284186X.2015.1004367 25734581

[28] Shaib WL Hawk N Cassidy RJ Chen Z Zhang C Brutcher E A Phase 1 Study of Stereotactic Body Radiation Therapy Dose Escalation for Borderline Resectable Pancreatic Cancer After Modified FOLFIRINOX (NCT01446458). Int J Radiat Oncol Bio Phys (2016) 96:296–303. 10.1016/j.ijrobp.2016.05.010 27475674

[29] Holyoake DLP Ward E Grose D McIntosh D Sebag-Montefiore D Radhakrishna G A phase-I trial of pre-operative, margin intensive, stereotactic body radiation therapy for pancreatic cancer: the ‘SPARC’ trial protocol. BMC Cancer (2016) 16:728. 10.1186/s12885-016-2765-4 27619800 PMC5020462

[30] Kharofa J Mierzwa M Olowokure O Sussman J Latif T Gupta A Pattern of marginal local failure in a phase ii trial of neoadjuvant chemotherapy and stereotactic body radiation therapy for resectable and borderline resectable pancreas cancer. Am J Clin Oncol (2019) 42:247–52. 10.1097/COC.0000000000000518 30724781

[31] Koay EJ Hanania AN Hall WA Taniguchi CM Rebueno N Myrehaug S Dose-Escalated Radiation Therapy for Pancreatic Cancer: A Simultaneous Integrated Boost Approach. Pract Radiat Oncol (2020) 13:S1879–8500(20)30035-7. 10.1016/j.prro.2020.01.012 PMC7423616 32061993

[32] Caravatta L Cellini F Simoni N Rosa C Niespolo MR Lupatelli M Magnetic resonance imaging (MRI) compared with computed tomography (CT) for interobserver agreement of gross tumor volume delineation in pancreatic cancer: a multi-institutional contouring study on behalf of the AIRO group for gastrointestinal cancers. Acta Oncol (2019) 58:439–47. 10.1080/0284186X.2018.1546899 30632876

