# Supplementary material for: Dosimetric Feasibility Study of Dose Escalated Stereotactic Body Radiation Therapy (SBRT) in Locally Advanced Pancreatic Cancer (LAPC) Patients: It Is Time to Raise the Bar
Source: Front Oncol. 2020 Dec 17;10:600940. doi: 10.3389/fonc.2020.600940 (PMC7773844; doi:10.3389/fonc.2020.600940)
Supplement: Supplementary file 1 [file DataSheet_1.docx]

**Supplementary Material**

**Table S1**. 15-fractions hypofractionated ablative radiation therapy (HART) vs 5-fractions dose escalated SBRT

|  | SBRT  (Gy/fr) | HART*  (Gy/fr) |
| --- | --- | --- |
| **PTV_t_** | 40 Gy (8 Gy)  BED_10_ 72 Gy | 52.5 Gy (3.5 Gy)  BED_10_ 71 Gy |
| **PTV_sib_** | 60 Gy (12 Gy)  BED_10_ 132 Gy | 84 Gy (5.6 Gy)  BED_10_ 131 Gy |
| **PTV_sip_** | 33 Gy (6.6 Gy)  BED_10_ 54.8 Gy  BED_3_ 105.60 Gy (EQD2 63.4 Gy) | 42 Gy (2.8 Gy)  BED_10_ 53.8 Gy  **BED_3_ 81.2 Gy (EQD2 48.7 Gy)** |

* The use of HART can allow to exploit the radiobiological principle of fractionation, allowing more permissive doses to OARs otherwise not obtainable with SBRT. Indeed, increasing the number of fractions, it's possible to reduce the BED (e.g. BED_3_ for PTV_sip_) to OARs, while maintaining higher effective total dose to the tumor and TVI.


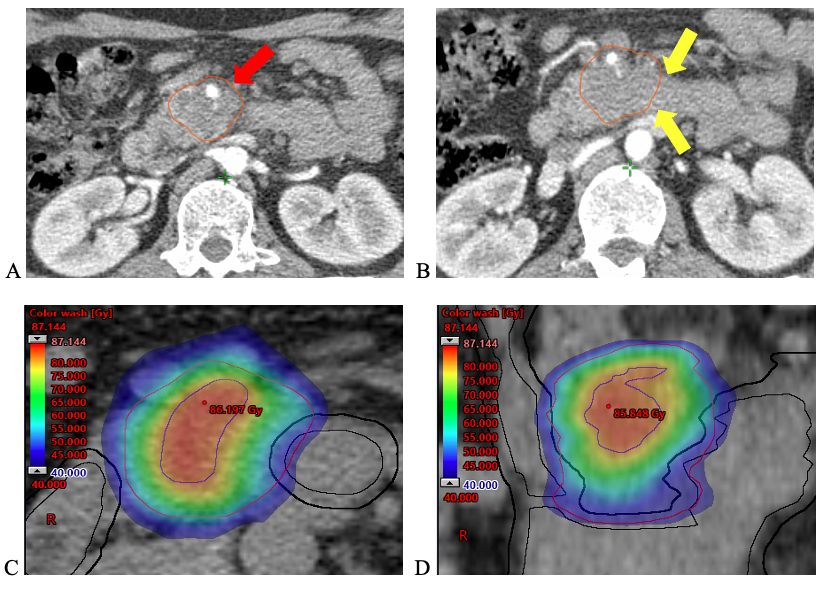


**Figure S1.** (A) and (B) Axial pancreatic CT-simulation phase images show large mass of pancreas, with encasement (red arrow) of superior mesenteric artery (SMA), with no cleavage plan/infiltration of duodenum (yellow arrows). (C) and (D) Dose distribution (color wash) for HART plan with Simultaneous Integrated Boost (SIB) and Simultaneous Integrated Protection (SIP). The prescription dose is 84/52.5/42 Gy in 15 daily fractions to PTV_sib_/PTV_t_/PTV_sip_, respectively. The sample plan demonstrates excellent PTVs coverage with appropriate respect of OARs.
